# The Importance of Tree Size and Fecundity for Wind Dispersal of Big-Leaf Mahogany

**DOI:** 10.1371/journal.pone.0017488

**Published:** 2011-03-07

**Authors:** Julian M. Norghauer, Charles A. Nock, James Grogan

**Affiliations:** 1 Faculty of Forestry, University of Toronto, Toronto, Ontario, Canada; 2 Département des Sciences Biologiques, Centre d'étude de la forêt, Université du Québec à Montréal (UQAM), Montréal, Québec, Canada; 3 Yale University School of Forestry and Environmental Studies, New Haven, Connecticut, United States of America; University of Zurich, Switzerland

## Abstract

Seed dispersal by wind is a critical yet poorly understood process in tropical forest trees. How tree size and fecundity affect this process at the population level remains largely unknown because of insufficient replication across adults. We measured seed dispersal by the endangered neotropical timber species big-leaf mahogany (*Swietenia macrophylla* King, Meliaceae) in the Brazilian Amazon at 25 relatively isolated trees using multiple 1-m wide belt transects extended 100 m downwind. Tree diameter and fecundity correlated positively with increased seed shadow extent; but in combination large, high fecundity trees contributed disproportionately to longer-distance dispersal events (>60 m). Among three empirical models fitted to seed density vs. distance in one dimension, the Student-*t* (2Dt) generally fit best (compared to the negative exponential and inverse power). When seedfall downwind was modelled in two dimensions using a normalised sample, it peaked furthest downwind (c. 25 m) for large, high-fecundity trees; with the inverse Gaussian and Weibull functions providing comparable fits that were slightly better than the lognormal. Although most seeds fell within 30 m of parent trees, relatively few juveniles were found within this distance, resulting in juvenile-to-seed ratios peaking at c. 35–45 m. Using the 2Dt model fits to predict seed densities downwind, coupled with known fecundity data for 2000–2009, we evaluated potential *Swietenia* regeneration near adults (≤30 m dispersal) and beyond 30 m. Mean seed arrival into canopy gaps >30 m downwind was more than 3× greater for large, high fecundity trees than small, high-fecundity trees. Tree seed production did not necessarily scale up proportionately with diameter, and was not consistent across years, and this resulting intraspecific variation can have important consequences for local patterns of dispersal in forests. Our results have important implications for management and conservation of big-leaf mahogany populations, and may apply to other threatened wind-dispersed Meliaceae trees.

## Introduction

Seed dispersal is a critical life phase for plants that plays a key role in driving the dynamics, distribution, and persistence of plant populations and communities [1]–[4]. This is especially true in tropical forests where most tree species are rare and thought to be severely seed limited [5]–[9]. Patterns of seed-fall set the stage for a suite of post-dispersal events that can affect recruitment rates, including seed predation and germination, herbivory and competition, resource use and acquisition, and overhead canopy disturbances [10]–[14]. Together, these events help structure tree population sizes and distributions, influencing the composition of adult forest trees at a given time and place [15]–[17], [3].

Local negative density-dependence (NDD) in juvenile tree recruitment and mortality is not uncommon in tropical forests, but likely stronger and more common in the critical seed-to-seedling and seedling-to-sapling stages (i.e., <1-cm stem diameter) [18], [19]. This NDD process is directly linked to the dispersal process because seeds dispersed relatively far should gain a fitness advantage by becoming locally rare [10], [11], [13]. Excellent empirical work has begun to examine interspecific variation in dispersal of forest trees [20]–[23], but less explored is intraspecific variation at the population level, especially for wind-dispersed trees representing c. 10–25% of species in these forests [6]. From both a conservation and management perspective, such research takes on greater urgency for threatened timber tree species.

A case in point is big-leaf mahogany, *Swietenia macrophylla* King (Meliaceae, henceforth ‘mahogany’), a high-value neotropical timber species that is both wind dispersed and threatened by overexploitation and deforestation [24]. This species has long been an iconic symbol at the nexus of sustainable forest management and conservation; it is on both IUCN's Red-List and CITES Appendix II because of regeneration failure and population declines after logging [25].

Tree regeneration can fail because of source disruptions to seed dispersal, which in turn can impact other ecological processes [3]. For timber species, the overriding disruption occurs via reductions in the seed source through removal of reproductive trees from the population. In the case of mahogany and related high-value Meliaceae, rampant logging has effectively ‘mined’ populations of large adult trees, exacerbating natural rarity in forests (<<1 adult trees ha^−1^ at landscape scales; [26]–[29]). At this scale mahogany has a very patchy non-uniform natural distribution such that, in places, it can occasionally have adult densities of >>1/ha, but these are the first to be logged out entirely. This risks regeneration failure by strengthening pre-existing seed and/or establishment limitations, which in turn may jeopardise local population persistence [5], [16], [20]. Additional mechanistic explanations for poor post-logging regeneration of mahogany in Mexican and Brazilian forests include insufficient light levels in the forest understory and logging gaps, and irregular supra-annual fruiting patterns by individuals and populations [30], [31].

Quantifying seed dispersal patterns in closed canopy forests is notoriously difficult [2], [6], [14]. The most common approach centers seed traps or transects on one or more adult plants, generating the data necessary to construct dispersal curves and estimate seed shadows (i.e., the spatial distribution of seed-fall) [32]. Alternatively, established seedlings/saplings can be surveyed to estimate the so-called ‘realized’ or ‘effective’ dispersal [4], [21], [33]. However, distributions of seeds and germinants are not likely identical, reflecting distance/density-dependence, microsite availability, and/or secondary dispersal effects upon recruitment success [14], [32]. Though several phenomenological models have been fit to such empirical data, in our view a critical yet overlooked issue is how parent tree size and fecundity affect the dispersal process, and what consequences this might have for local demography.

Several intrinsic species' traits influence seed crop sizes and spatial distribution patterns of plant dispersal (i.e., the seed shadow) [1], [8], [16], [32], [35], [36]. These include seed mass and morphology, stem diameter and/or height distributions, plant architecture, crown area and exposure, as well as how propagules are distributed within crowns [4], [6]. But trees are long-lived organisms, and a population of adults will have significant variation in diameters, heights and crown volume/area ratios across individuals, and in their annual and lifetime fecundity [17], [34], [35]. These attributes all shape the scale and extent of seed shadows in forests, yet their relative contribution to the dispersal process within populations is less clear.

Because the true source of dispersed seeds is often unknown, an inverse modelling approach may be used that assumes fecundity scales positively with tree diameter, or by extension, stand basal area [2], [20], [21], [23], [33]. While a positive diameter-fecundity relationship may be reasonable over the entire lifetime of trees – for example, mahogany trees >70 cm diam are more fecund than smaller individuals [30], [31] – there is little empirical evidence to support this notion for trees in the short term, probably because (1) differences in seed and flower production may be heritable; (2) changing stem diameter/crown area allometry is a poor predictor of primary productivity; (3) variable fruiting and annual crop sizes are unpredictable and may be resource and/or pollinator limited [34]–[36]. For these reasons, a larger sample of source trees than is typically used (≤5) is needed for investigating seed dispersal variation within populations [4].

Three methodological steps can deepen our understanding of dispersal. First, sampling should be extended well beyond where most seeds cluster near parent trees to detect long distance dispersal events (LDD) [2]. Second, previously established seedlings should be sampled in addition to dispersed seeds to detect post-dispersal changes to the seed shadows that may result from NDD processes. Third and most importantly, replication should be sufficient across a range of reproductive adults varying in size and fruit production [4]. From a management perspective, these steps are critical for better understanding the spatial distributions of advance understory regeneration before and after harvesting, and for selecting seed trees. Both aspects are crucial for conserving remnant mahogany populations.

In this study we documented intraspecific variation in seed dispersal around 25 relatively isolated fruiting trees in a logged mahogany population in Pará, Brazil. We asked: (1) What is the relationship between tree traits, namely stem diameter and fecundity (seed crop size), and patterns of seed dispersal? (2) Which phenomenological model(s) best describes the relationship between distance and density of dispersed seeds? (3) What is the relationship of already established mahogany seedlings to observed seed-fall patterns? (4) What are the possible consequences of dispersal variation for spatial patterns of adult recruitment? We end by considering the implications of (1) – (4) for management and conservation of this rare and threatened tree species and related genera. As far as we know, this sample size is among the largest for describing seed dispersal of any forest tree.

## Methods

### Study species

Big-leaf mahogany is a canopy-emergent tree sometimes exceeding 50 m height and 2 m diameter with an extensive natural range from Mexico to Bolivia [26]. Adult trees are monoecious with reproductive onset from c. 20–30 cm diam, though fruit production is rare <30 cm diam [27], [30], [31]. Fruit production is annual or supra-annual. Woody fruit capsules are 9–19 cm in length and contain c. 40–42 viable seeds per fruit [31]. Seeds are large for a wind-dispersed species (mean wet/dry weight  = 0.56 g/0.37 g) and encased in 5–13 cm long cinnamon-coloured, winged samaroid diaspores that are conspicuous in the leaf litter layer [31]. In the southeastern Amazon region, mahogany seeds disperse during the early to mid dry season; dispersal is nearly complete by early September, and is strongly skewed to the west of parent trees by prevailing dry-season winds [31], [37]. Similar results have been reported from Mexico [38]. Seeds lack a dormancy mechanism; germination is triggered by moisture imbibition during the early wet season months [31].

### Study site

Our study was conducted in a 704-ha area within a larger forest fragment (4100 ha) called Marajoara (7°50′S, 50°16′W) located in southeast Pará state, 34 km northwest of Redenção. This forest was selectively logged in 1992–1994 for mahogany, reducing landscape-scale density from 0.65 to 0.19 trees >20 cm diam ha^−1^ [28]. Forest vegetation is semi-evergreen with a deciduous component. Climate is tropical dry and strongly seasonal, with rains (c. 1600–2100 yr^−1^) falling primarily between September and May [39].

### Measuring seed dispersal

On 13–14 August 2005 we selected 25 mahogany trees >30 cm diam within the study area that had completed seed dispersal or were near completion — that is, dehisced fruit capsules in the crown appeared almost bare. These trees were selected to maximise interspersion across the study area; all mahogany trees were separated from all fruiting or non-fruiting live conspecific trees by at least 125 m. During the next two weeks, six 1-m wide belt transects were established at 60° intervals radiating out from the base of each tree on the following bearings: NE (30°), E (90°), SE (150°), SW (210°), W (270°), and NW (330°). Transects extended 100 m downwind of each tree (i.e., SW, W, and NW) but only 50 m upwind (i.e., NE, E, and SE) because few seeds were expected on the windward side [31]. All transects were carefully searched for mahogany seeds, first by examining the litter surface and then carefully sifting through the leaf litter. The number of intact or predated seeds per 5-m distance interval was noted. We also noted the presence of live mahogany juveniles that established in the previous year (2004) or earlier (any stems c. 18–20 cm tall and upwards) in each 5-m interval and recorded stem height to the nearest cm.

We used multiple transects because it was impractical to thoroughly search ‘wedges’ at so many trees (n = 25) needed to investigate variation in dispersal at the population level. Wedges that sample a constant proportion of the annulus with increasing distance reduce the risk of underestimating dispersal events at far distances (the tail). However, Skarpaas et al. [40] showed that when the prevailing direction of dispersal *is already known*, radial transects of a fixed width can describe the true seed shadow just as well as sampling in wedges.

Lateral tree crown extensions were estimated for each sampled tree by moving along each of the six bearings measuring the distance at which the overhead crown edge was perpendicular to the ground. For a given tree, the end-points of the crown extensions were connected with straight lines to form a six-sided polygon and the six triangles therein were summed to estimate the crown cross-sectional area (m^2^). We already knew that mahogany's cross-sectional crown area increases exponentially with tree diameter, whereas height to the base of the crown increases linearly with diameter (see Grogan 2001 cited in [31]).

### Measuring tree seed crops

**Figure 1:**
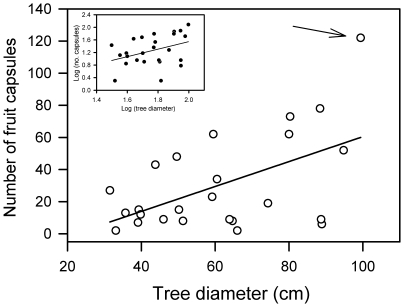
Fruit production as a function of stem diameter for 25 mahogany (*Swietenia macrophylla*) trees in 2005 in Pará, Brazil. One fruit capsule contains on average 40–42 viable seeds. The regression line was significant (adj. *r* ^2^ = 0.26, *P* = 0.0058, *y* = –17.1+0.775*x*) but lacked constant variance. The arrow points to a heavy fruiter outside 95% confidence intervals; its removal from the analysis resulted in a marginally significant relationship between tree diam and fruit production (adj. *r* ^2^ = 0.13, *P* = 0.047). The inset shows the same 25 trees with diam and reproductive output log-transformed to stabilise the variance (adj. *r* ^2^ = 0.14, *P* = 0.0607).

To directly measure tree-level seed production (henceforth ‘fecundity’), two separate observers counted fruit capsules within the crown of each sampled tree. Mahogany's large woody fruit capsules are readily identifiable in the crown from the ground during the mid to late dry season when crowns are leafless or nearly so. Each capsule consists of five pericarps that break apart and drop off in the dry season to expose the winged diaspores. To verify these tree-level fruit counts, fallen dehisced capsule pericarps (5 = 1 fruit) were collected beneath the crown area of each fruiting tree and the total count divided by five. The final capsule count used in analyses as a measure of individual tree fecundity was the larger figure yielded by the two methods (**Fig. 1**). This fecundity value estimated source strength (number of seeds released) in analyses.

### Tree seed crops estimated from transects

For each 5-m annulus, total seed counts in the six radial transects were converted to seed density (/m^2^) by dividing by 30 m^2^ (assuming no seeds landed further than sampled distances on upwind transects [31]) and multiplied by 1/3 the annulus area to obtain seed count per annulus. These new counts were summed over the 20 annuli to yield an estimate of tree seed production. The 1/3 correction corresponds to the 120° (out of 360°) downwind arc where most seeds fall during dispersal because of strong anisotropy. The key assumption in this calculation is that seed densities in our belt transects were accurate and sufficiently representative of true densities downwind. These scaled-up *indirect* estimates of trees' seed crops closely matched direct estimates from the census of fruit capsule production described above (*r* = 0.85; log-transformed values, *r* = 0.89, n = 25 trees), but were underestimated for trees with >40 capsules (points begin to fall below 1∶1 line).

### Tree size-fecundity groupings

To investigate how mahogany seed shadows might change as a function of parent stem diameter and fruit crop size, we grouped sampled trees into four size-fecundity categories using the commercial diameter size threshold of 60 cm to distinguish ‘small’ from ‘large’ trees. The median value of fruit capsule production in 2005 (15 capsules) for the sample population of 25 trees was used as the threshold distinguishing the two fecundity classes. Categories and sample numbers were: **small** tree size with **few** fruits (**SF**, 30–60 cm diam, ≤15 capsules, n = 8); **small** size with **many** fruits (**SM**, 30–60 cm diam, >15 capsules, n = 5); **large** with **few** fruits (**LF,** 60–100 cm diam, ≤15 capsules, n = 5); and **large** with **many** fruits (**LM**, 60–100 cm diam, >15 capsules, n = 7). For a subset of LM trees lacking fruiting conspecific(s) within 200 m (n = 4) the downwind search was extended by widening transects to 3 m from 100–160 m. The rationale here was to take advantage of this greater intraspecific tree spacing to detect long distance dispersal (LDD) events by doubling our sampling effort.

### One-dimensional model fitting

Parent tree stems and not crown edges were used as the point sources for all modelling of dispersal. Three well known, one-dimensional functional forms of dispersal were tested for their fit to the mahogany seed density vs. distance data (**Table 1**): (1) the negative exponential model (NE); (2) the inverse power model (IP); and (3) the Student-*t* model (2Dt). For descriptions and derivations of these models, see references [4], [20], [41].

**Table 1:** Empirical models tested for mahogany (*Swietenia macrophylla*) seed dispersal expressed in one dimension and two dimensions downwind of 25 isolated trees at the Marajoara forest in Pará, Brazil.

Function	Equation
*One-dimensional fitting* 1	
NE	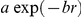
IP	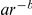
2Dt^2^	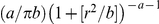
*Two-dimensional fitting* 3	
LN^4^	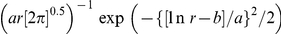
IG^5^	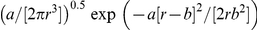
WB^6^	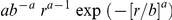

*r* is the distance from the stem of the source tree.

1 Cousens et al. [4] (see Table 5.2 therein).

2 see Clark et al. 1999 [20].

3 Cousens et al. [4] (see Table 5.1 therein).

4 see Greene et al. 2004 [21].

5 see reference of Whelan 1988 in [4]; this is not the modified Wald equation by Katul et al. 2005 in which both parameters are estimated from wind speed and plant attributes.

6 see Higgins & Richardson 1999 in [4]; also see [21] and [33].

Following the widely used approach of Bullock & Clarke [41], and the notation used by Skarpaas et al. [42], the seed shadow in one dimension is expressed as (*s*) (seeds/m^2^), with total seed count (*c*) at distance *r* estimated as:
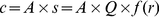
(1)


In this equation, *A* is the trap area; *Q* is the number of seeds released, or source strength; *r* is the radial distance, and *f*(*r*) is the dispersal model (1/m^2^) described by equations listed in Table 1. In our mahogany study, *A* is the always same ‘trap’ area of 15 m^2^ maintained at each distance *r*. The *c* is total seed count downwind of each tree summed over SW, W, and NW transects, also at distance *r*. The *Q* is an individual tree's seed crop, estimated as 40 seeds × no. of its fruit capsules. The *f*(*r*) models fitted are shown in top part of **Table 1**.

Because we knew in advance that mahogany dispersal is skewed west of trees, a one-dimensional analysis was justified because it describes seed dispersal in a single direction [4], [32]. Others have applied this approach to plants when strong anisotropy is already known [40]–[43]. In doing this, however, we actually assumed that the dispersal patterns in the centre transect (W) vs. the edge transects (SW, NW) were similar; that is, we assumed uniformity between the three transects, which was not the case for all trees in the sample (data not shown, more seeds go W > NW > SW; see below Results, also see [31]).

### Two-dimensional model fitting

Another problem with a one-dimensional analysis, as described above, is that it fails to account for the widening of the seed shadow downwind, and the decreased probability to land in a given area as the distance gets farther. This can be addressed by modelling dispersal in all possible directions using a two-dimensional fitting approach in the form of a probability density function (pdf). The latter can describe either the probability of distance occurring in any direction, based on a histogram of absolute counts, or the probability of a given propagule landing at an infinitely small distance *r* from the parent (the latter is simply the histogram of counts' density divided by total seeds dispersed, i.e., *Q*)[4]. The key point here is that a one-dimensional approach accounts only for the variability along the radial direction, whereas a pdf tries to account for the distribution along the axial direction as well.

To generate a pdf, for each transect, to 100 m, we first multiplied the number of seeds sampled in each 1-m wide ×5-m long ‘trap’ segment by a scaling factor of π(2r–5)/30. To avoid pseudo-replication, these new *normalised sample* counts were first averaged across the three downwind transects (SW, W, NW) for each 5-m annulus, then divided by their sum. This yielded a relative frequency of dispersal distances attained downwind (a pdf). We caution, however, that this normalisation of sampling area involves scaling up of integers, for which values of zero stay zero [41]. Three well-known models were fitted to normalised frequency distribution of dispersal distances: the lognormal (LN), the inverse Gaussian (IG) and the Weibull (WB), as shown in the lower part of **Table 1**; however, not as part of eqn. (1) where only seed density vs. distance was modelled instead. Finally, pdf and seed density in one dimension are readily interchangeable if one assumes dispersal is isotropic [4], which we cannot for mahogany. However, a pdf could still be applied for comparative purposes, as done here, because it sums to unity (i.e., 1), and thus should control for fecundity effects.

### Statistical methods

We used one-way analysis of variance (ANOVA) to compare tree traits across the four size-fecundity groups. Three of the four dependent variables were transformed to stabilize variances and normalise model residuals. Specifically, both the number of fruit capsules and tree crown area were log-transformed, whereas tree crown area to diameter ratio was square-root transformed. One-way non-parametric analyses (Kruskal-Wallis tests) were used to compare patterns of seedfall among the four tree groups because neither log nor arcsine-square transformations met ANOVA assumptions based on visual inspection of the residuals against predicted variables.

We used the maximum likelihood method and default Nelder-Mead algorithm in the ‘mle2’ function of R (package ‘bbmle’ [44]) to estimate parameter values when fitting the three one-dimensional functions to the seed density vs. distance data for each of the four tree groups (SF, SM, LF, LM). A Poisson error distribution was assumed for seed counts following studies of other wind-dispersed tree species [20], [21], [33], [40]–[43]. Models were evaluated using the negative log-likelihood (–ln *L*) and Akaike's Information Criteria (AIC); for each the lowest value indicated the best overall fit. The above was also used for the normalised seed counts modelled in two dimensions.

Stepwise multiple linear regressions using a backward elimination process were performed to determine which tree traits best predicted total number of seeds downwind and their mean dispersal distances, and to gauge seed shadow extent and total number of seeds exceeding 60 m distance downwind (SW, W, NW). Dependent variables were based on a *normalised sample* (see above scaling factor) in two dimensions to estimate overall seed counts had a constant proportion of annulus been sampled instead of a diminishing one with distance. In these analyses, to satisfy model assumptions, all dependent variables were log-transformed as were the predictors tree crown area and total number of fruit capsules, whereas tree diameter and height were not. Model residuals and final model predictor variables were examined visually for heteroscedasticity and for collinearity using tolerance values and variance inflation factors (i.e., VIF  = 1/tolerance); tolerance values <0.1 and VIFs >10 point to strong collinearity [45].

To avoid pseudo-replication, the heights of multiple juveniles found in the same 1-m ×5-m segment of a radial transect were first averaged prior to analyses. The relationship between patterns in seedfall and juveniles downwind of trees was investigated using the mean ratio of juveniles to seeds across the 25 trees. The number of juveniles across the SW, W, and NW transects were averaged on a per tree basis, as were the number of seeds in a given distance interval, based on their normalised counts (see above for ‘scaling factor’ used). Beyond 65 m, however, the total sample sizes from transect sampling became very small and thus prone to disproportionately greater error when scaled upwards (6–23 seeds per distance, and 8 juveniles only). For this reason we omitted data at these farthest distances. We fit a Loess smoother (parameter  = 0.3) to explore possible changes in recruitment with ontogeny because it is more robust to this declining precision of the ratio with increasing distance from the source tree [45].

### Estimating seed arrival in gaps near and far from trees

Seed densities per distance predicted by 2Dt (for LF, SF and LM, SM respectively) were converted into proportions of total seed crop in a 120° arc downwind. To estimate seed abundance near (≤30 m) and beyond >30 m downwind, the proportions of seed per downwind annulus segment were multiplied by each tree's actual seed crop for a given year (2000–2009, see **Supporting Information S1**) assuming 40 viable seeds per fruit capsule. These seed abundances were then averaged across sampled trees on a yearly basis for each of the four size-fecundity groups, and each multiplied by the forest area (4%) likely in a ‘gap-like’ state when or soon after dispersal occurred. In the 1996–1997 wet season 2.6% of 16.5 km of trails at the site had new gaps formed because of tree- and branch-falls (<2 m tall vegetation, see [39] for details). Because the majority of these newly formed gaps do not fill in with vegetation within a year's time, we assumed that half (1.3%) might be suitable for mahogany growth in the next year (a reasonable assumption in our experience, 1.3%+2.6%  =  ∼4%).

## Results

### Dispersal pattern

We found 5154 winged diaspores (hereafter, seeds) in transects around 25 mahogany trees (**Table 2**). Of these, 3622 seeds (70.3%) were found in 100-m long downwind transects west of parent trees (SW: 19.8%, W: 27.8%, NW: 22.7%), and 1521 seeds (29.7%) were found in upwind transects east of parent trees. Of these upwind seeds 78% were found within 10 m of the tree trunk, with hardly any landing beyond 20 m. Downwind seeds were located in all distance classes, with a peak count of 62 seeds (12.4 seeds m^−2^) in a NW transect at 0–5 m distance. At four trees searched beyond 100 m, the maximum distance travelled by a found seed was 155 m. This occurred in the W transect of the tree with the highest fruit production (122 capsules).

**Table 2:** Seed dispersal patterns of mahogany (*Swietenia macrophylla*) trees (n = 25) at the Marajoara forest in Pará, Brazil.

		Total no.seeds found	No. seeds	No.seeds	Estimatedtotal seed cropper tree	Proportion of estimated total seed crop per tree			No. seeds>50 m in transects
Tree group	n	All six transects	Easttransects	Westtransects		<15 m	15–50 m	>50 m	
SF	8	91±24	30±12	60±13	362±70	0.38±0.06	0.45±0.05	0.17±0.06	69±24
SM	5	306±57	118±27	188±46	1067±159	0.47±0.09	0.39±0.06	0.14±0.05	155±54
LF	5	97±33	44±32	53±4	445±95	0.33±0.07	0.40±0.03	0.26±0.08	97±37
LM	7	345±98	68±23	276±76	2263±554	0.19±0.03	0.56±0.01	0.25±0.02	542±107
χ^2^		13.0***	8.6**	16.7****	17.4****	9.5**	7.2*	4.3	13.4***

*Notes* : The categories are combinations of small vs. large tree sizes (30–60 cm vs. 60–100 cm diam, respectively) with few vs. many fruits produced (≤15 capsules vs. >15 capsules, respectively [size-fecundity classes: SF  =  small–few, SM  =  small–many, LF  =  large–few, LM  =  large–many]). Values presented are means ± (1 SE). Within column comparisons across tree groups were made using Kruskal-Wallis tests (df = 3).

*P<0.1,

**P<0.05,

***P<0.005,

****P<0.001.

### Dispersal patterns across tree groups

Sampled trees differed greatly by individual size and fecundity (**Table 3**). Tree diameters ranged threefold, from 32–99 cm diam. Estimated tree crown areas ranged 15-fold, from 37–612 m^2^, while fruit crop size ranged from 2–122 capsules. Differences in crown area and the ratio of crown area to diameter were not as pronounced, with SM and LF trees sharing similar values. Crown area was positively correlated with tree heights (Pearson *r* = 0.74; missing heights for 10 of 25 trees were interpolated).

**Table 3:** Tree characteristics of mahogany (*Swietenia macrophylla*) trees grouped into four size-fecundity classes at the Marajoara forest in Pará, Brazil.

Variables	*F*_3, 24_	SF (n = 8)	SM (n = 5)	LF (n = 5)	LM (n = 7)
1Diameter (cm)	21.29**	41.8±2.3^a^	48.7±5.2^a^	74.4±5.9^b^	82.5±4.9^b^
No. capsules†	17.05**	10.1±1.6^a^	40.6±7.1^b^	6.8±1.3^a^	63.1±12.7^b^
2Height (m)	17.77**	21.8±0.6^a^	24.0±0.7^a^	27.6±1.2^b^	28.1±0.7^b^
Crown area (m^2^) †	14.32**	59.0±6.7^a^	95.0±26.4^ab^	157±10.2^bc^	253±48.9^c^
Crown area: diam ‡	4.47*	1.40±0.17^a^	1.91±0.48^ab^	2.15±0.18^ab^	3.07±0.55^b^

*Notes* : Means (± SE) with different letters are significantly different at α = 0.05 following post-hoc Tukey HSD tests [size-fecundity classes: SF  =  small diam–few fruits, SM  =  small diam–many fruits, LF  =  large diam–few fruits, LM  =  large diam–many fruits].

One-way ANOVA model significance:

** *P*<0.0001,

* *P* = 0.014.

1 Measured in 2005.

2 Height to the base of the live crown; measured in 1998 on 15 of the 25 trees, with other 10 interpolated using regression of height vs. diam (n = 153 trees).

† log-transformed variable for ANOVA.

‡ square-root transformed variable for ANOVA.

**Figure 2:**
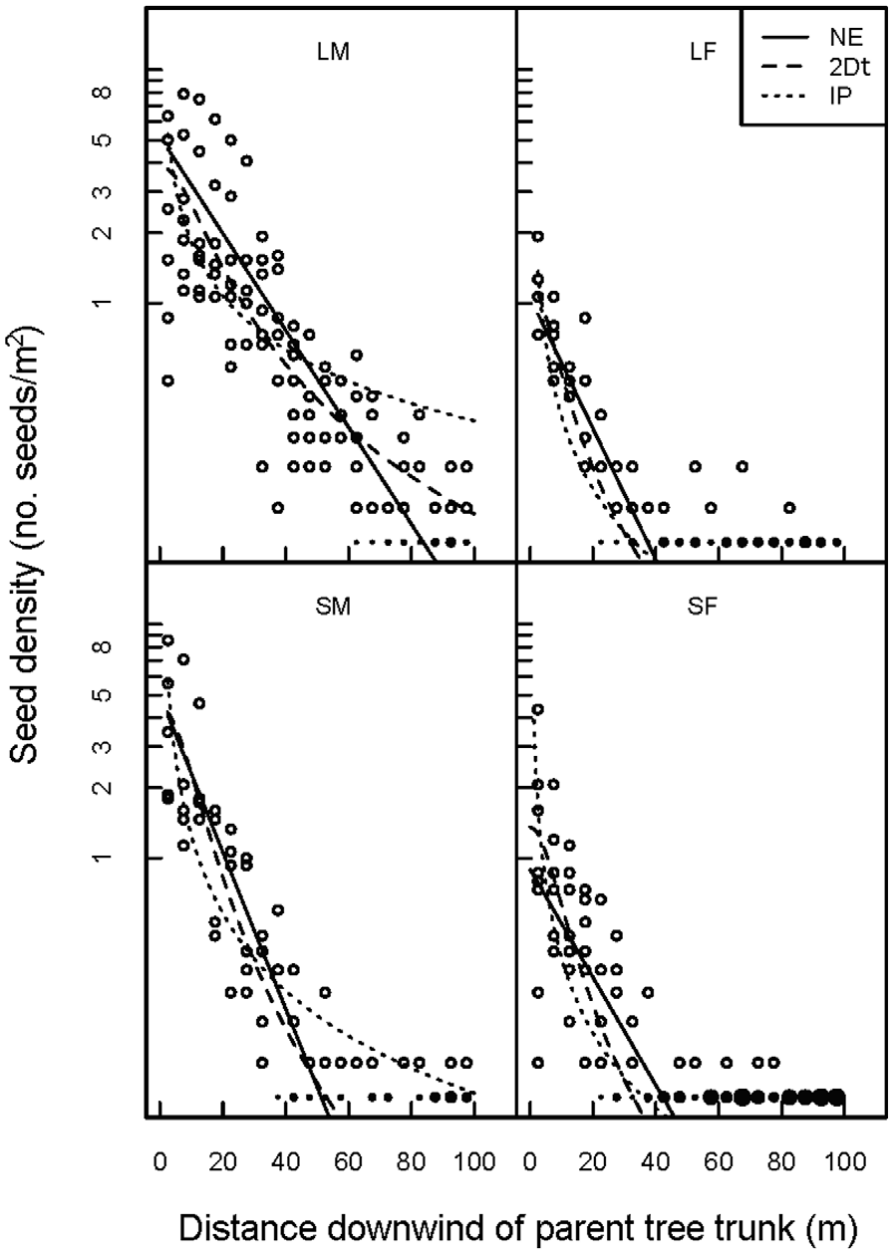
Seed densities of mahogany (*Swietenia macrophylla*) dispersal downwind from 25 parents in Pará, Brazil. The isolated parent trees were assigned to one of four groups based on diam and fruit crop size: SF  =  small–few, SM  =  small–many, LF  =  large–few, LM  =  large–many. Open circles show overall seed densities in 1-m ×5-m quadrats along W, NW, and SW 100-m long transects; filled circles represent zero seed densities, with symbol size proportional to frequency, observed along these same transects. Curves shown are fitted functions: NE  =  negative exponential; 2Dt  =  Student-*t* distribution; and IP  =  inverse power (see **Table 1**). Note the logarithmic scale on y-axis.

In transects, we found three times as many seeds around trees producing many (M) vs. few (F) fruit, irrespective of tree size, and these more fecund trees had more seeds dispersed downwind as well (**Table 2**). While >50% of transect seeds were found within 15 m of tree stems for all tree groups, in terms of total estimated seed crop, on average, the lowest percentage (19%) of seeds dispersed within a 15-m radius was observed at large trees producing many fruits (LM). A significantly higher proportion of LM seeds landed 50–100 m downwind compared to the other three groups (**Figs. 2** **,** **3**). In sum, SM, SF, and LF showed relatively similar seed dispersal patterns compared to LM trees, which spread seeds out more and attained longer dispersal distances (**Figs. 2** **,** **3**).

**Figure 3:**
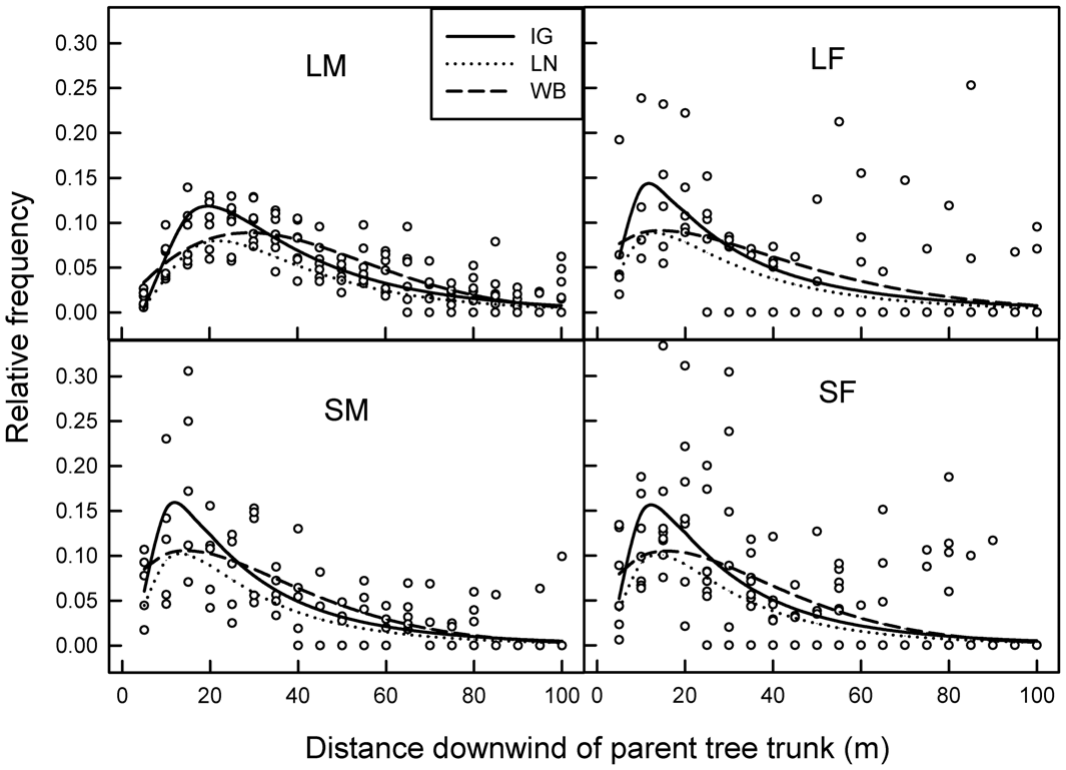
Dispersal of mahogany (*Swietenia macrophylla*) seeds in two dimensions downwind of 25 parent trees in Pará, Brazil. Relative frequency of dispersal is the proportions of normalised seed counts downwind per tree for a given 5-m distance interval, generated from the original 1-m wide transects. The probability density function (pdf) of a given distance interval occurring was fitted to the normalised data using three equations: IG  =  inverse Gaussian (Wald); LN  =  lognormal; and WB  =  Weibull (see **Table 1**).

### Evaluation of dispersal functions

The 2Dt provided the best overall fit for dispersal of small trees irrespective of fecundity (SF, SM), and for large trees with few fruits (LF). For LM trees the 2Dt also fit reasonably well, but not as well as the NE function. The IP consistently fit poorly for all four combinations of tree size and fecundity. The predicted 2Dt curve of LM trees was different from the SM, LF, and SF trees in having a longer tail (**Table 4**); the latter two were very similar for all functions fitted (**Fig. 2**). Similar results — namely the broader, ‘fatter-tailed’ seed shadow of LM — were found when dispersal was expressed as the relative frequency of dispersal distances occurring, and modelled as a pdf using LN (lognormal), IG (inverse Gaussian) and WB (Weibull) fitted functions (**Fig. 3**). Compared to **Fig. 2**, modelling dispersal in two-dimensions made LF, SF, and SM dispersal patterns more similar, with a peak now revealed in seedfall beginning at c. 5–10 m from the tree base, but furthest in LM trees at c. 25 m. The IG and WB fit comparably well, and slightly better than LN, for this species at the site.

**Table 4:** Estimated parameters and fit statistics of empirical dispersal models for 25 mahogany (*Swietenia macrophylla*) trees at the Marajoara forest, Pará, Brazil.

	*One-dimensional model fitting*					*Two-dimensional model fitting*				
Tree group	Function	*a*	*b*	–ln *L*	AIC	Function	*a*	*b*	–ln *L*	AIC
Small–few (SF)	IP	0.012	1.069	335.8	675.6	LN	1.062	3.206	34.7	73.4
	NE	0.002	0.053	290.0	584.1	**IG**	43.650	32.343	**32.4**	**68.8**
	**2Dt**	1.050	92.635	**287.2**	**578.3**	WB	1.462	35.228	32.6	69.1
Small–many (SM)	IP	0.010	1.101	447.7	899.5	LN	1.086	3.185	21.6	47.2
	NE	0.003	0.077	336.9	677.8	**IG**	41.230	32.128	**20.2**	**44.3**
	**2Dt**	1.291	149.940	**327.4**	**658.8**	WB	1.415	34.800	20.3	44.6
Large–few (LF)	IP	0.014	1.032	188.0	380.0	LN	1.194	3.298	22.6	49.2
	NE	0.004	0.064	175.5	354.9	**IG**	38.349	37.848	**21.1**	**46.3**
	**2Dt**	0.871	57.328	167.5	**338.9**	WB	1.343	40.282	21.2	46.4
Large–many (LM)	IP	0.004	0.766	878.7	1761.5	LN	0.918	3.512	31.1	66.2
	**NE**	0.002	0.047	**554.6**	**1113.2**	IG	77.146	40.796	29.2	62.3
	2Dt	0.843	174.179	601.0	1206.0	**WB**	1.804	45.293	**29.0**	**62.0**

Better-fitting models indicated in bold.

*Notes:* Tree groups are factorial combinations of small vs. large tree size (30–60 cm vs. 60–100 cm diam, respectively) and few vs. many fruits (≤15 capsules vs. >15 capsules, respectively. Differences in AIC greater than 2 indicate little evidence in support of competing/alternative models. See Table 1 for equations describing the dispersal functions.

### Long-distance dispersal (LDD)

We found 40 mahogany seeds 100–160 m downwind of four LM trees (8, 7, 9, and 16 seeds tree^−1^ in a pooled sampled area of 2160 m^2^), equivalent to 0.0185 seeds m^−2^. We occasionally came across multiple seeds (2–4) in the same 3-m ×5-m quadrat, or clumped among two consecutive quadrats along transects, likely carried there together by strong uplifting winds [31], [46].

Multiple step-wise regression analyses showed that none of the four traits significantly predicted mean seed dispersal distance downwind, except for possibly tree crown area at a less stringent α = 0.10 (*P* = 0.086; model *F*_1, 23_ = 3.22, *R* ^2^ = 0.12). However, the number of fruit capsules (*P*<0.0001), and to a lesser extent, crown area (*P* = 0.0034), explained 87% of variation in the total number of seeds landing downwind of mahogany trees (model *F*_2, 22_ = 72.4, *R* ^2^ = 0.87). Similarly, the number of seeds that exceeded 60 m distance – a proxy for seed shadow extent – was best predicted by number of fruit capsules (*P* = 0.0007) and crown area (*P* = 0.024) (model *F*_2, 22_ = 17.3, *R* ^2^ = 0.61). Neither tree height nor diameter was a significant predictor in the three models (all six *P* values = 0.35–0.98).

### Comparing seed distributions to existing juveniles

**Figure 4:**
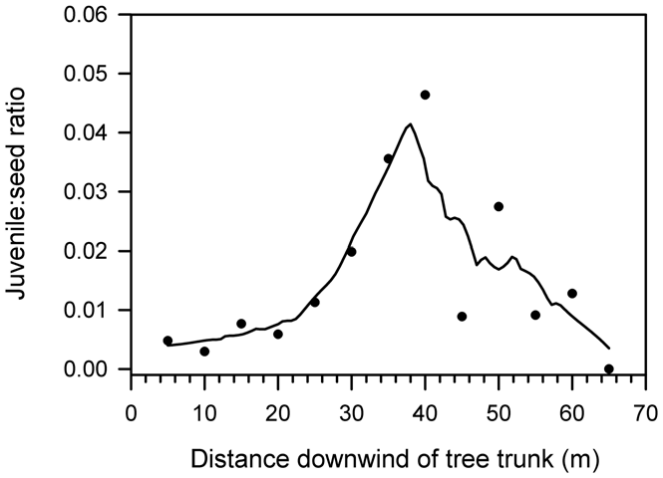
The ratio of live mahogany (*Swietenia macrophylla*) juveniles to dispersed seeds downwind of 25 parent trees in Pará, Brazil. A Loess smoother line was fit to guard against diminished precision with increasing distance in 5-m intervals (see Methods for details). Data shown are limited to <70 m distance because of very low number of seeds and high degree of imprecision at distances thereafter.

A total of 82 mahogany juvenile stems were found in 11150 m^2^ around 25 adult trees. Not a single large juvenile (>50 cm tall) was found beneath the canopy of any sampled tree; the largest juvenile encountered was 80 cm tall, 70 m downwind of a small (50 cm diam) tree. Only 4 juveniles were found more than 15 m from any parent on upwind transects (NE, E, SW; total upwind  = 16). Of the 66 juveniles at downwind distances, 18%, 48%, and 33% of them were found in SW, W, and NW directions, respectively. Mean log-transformed heights (± SE) of juveniles were significantly different among distance classes (one-way ANOVA, *F*_2, 69_ = 4.13, *P* = 0.0201). Juveniles farther away (50–100 m) were significantly taller (∼25% more) than juveniles within 50 m of parent trees (means for respective distance classes: 0–15 m, 28.5±1.9 cm; 15–50 m: 31.0±2.1 cm; 50–100 m: 41.5±5.3 cm). The ‘repulsion effect’ of parent trees on offspring, defined here as the juvenile-to-seed ratio, increased with dispersal distance and peaked at 35–50 m (**Fig. 4**).

### Potential regeneration near and far from trees

**Figure 5:**
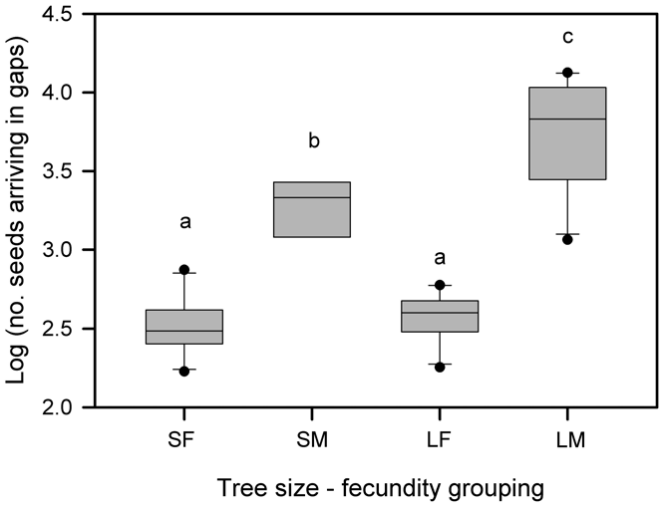
Mahogany (*Swietenia macrophylla*) seeds expected to land in canopy gaps far (>30 m) downwind of 25 parent trees in Pará, Brazil. Trees were grouped into small and large trees (<60 cm diam vs. ≥60 cm diam) with few or many fruits (≤15 vs. >15 capsules) estimated using fecundity data during 2000–2009 (see Supporting Information S1). Shown are boxplots for each size-fecundity group based on 10, 8, 10 and 10 years of fruit production data per class (SF, SM, LF and LM, respectively). Means (± SE) were 4.42±0.64, 25.1±3.3, 4.97±0.51, and 84.3±16.7, respectively. Significant differences among groups are shown by different lowercase letters, using an averaged LSD  = 0.221 based on a significant size × fecundity interaction in an unbalanced repeated measures ANOVA with year as a blocking factor: *F*_1, 25_ = 7.47, *P* = 0.011. The blocking effect was not significant (*P* = 0.36) while main effects were.

For the size-fecundity groups SF, LF, SM, and LM the mean total seed inputs ≤30 m downwind, in a 120° arc, ranged from 49–218, 39–130, 443–1189, and 171–1976 seeds per year, respectively. However, seed arrivals into canopy gaps beyond 30 m, where adult recruitment is more likely (**Fig. 4**), was more than 3x greater for LM than SM, with both groups exceeding inputs downwind generated by SF and LF trees (**Fig. 5**).

## Discussion

### Mahogany's seed shadow

Most big-leaf mahogany seeds dispersed to the west or downwind, yet the bulk of them landed beneath or near crowns (<30 m). This result agrees with two previous studies of 6 and 11 heavily fruiting big-leaf mahogany adults, at Marajoara and in Mexico, respectively [31], [39], but not in a Bolivian forest [27] nor for the closely related *Entandrophragma* spp. (African mahogany) [47]. This anisotropic dispersal was highly predictable despite wide variation in crown areas and branching patterns. The most plausible explanation is that, coupled with mahogany's emergent position above a low irregular canopy, pervasive, strong local dry season winds generate this pattern each year. Similar skewed seed deposition patterns were observed for *Lonchocarpus pentaphylus* (n = 1) and *Tachigalia versicolor* (n = 2) trees in a Panamanian forest [48], [49]. Whether local winds skew dispersal in temperate forest stands remains less clear because most dispersal studies assume a symmetric two-dimensional kernel when model fitting, which is increasingly questionable for wind-dispersed species [6], [50]. Nonetheless, many studies of dispersal in temperate stands confirm the general impression of very localized seedfall (irrespective of vector), except for perhaps the smallest-seeded species [16], [20], [21], [51]. In tropical forests, where stands are more mixed and structurally diverse, a steep decline of seed density with distance from the parent tree is the norm rather than the exception [1], [13], [17], [22], [23].

### Tree size-fecundity effects on seed dispersal

In this study mahogany reproduction was only weakly predicted by tree diameter, and was highly variable within individuals and across 10 years (**Fig. 1**, **Supporting Information S1**). In a given year only a few trees will have large seed crops, and some skip reproduction altogether. Large successive crops of fruit on the same tree are rare, a pattern likely for many other tropical trees in other forests as well [34], [35], [51]–[55]. In an African rain forest, fecundity was poorly explained by individual tree traits like crown area, height and diameter for nine species including three that were wind-dispersed [22]. Elsewhere, diameter was a significant yet imprecise (*r* ^2^ = 0.26) predictor of tree-level fecundity in only 8 of 14 tree species in a Malaysian rain forest [56]. Hence the generality of using a positive scaling of diameter with fecundity deserves greater scrutiny when modeling dispersal within a species if tree size-fecundity relationships tend to flatten beyond a reproductive size-threshold [51].

Small mahogany trees with many fruits (SM) cast a slightly broader seed shadow than did large trees with few fruits (LF), but both were eclipsed by large trees bearing many fruits (LM; **Figs. 2** **,** **3**, **Table 4**). This suggests that producing more fruits is more effective to a parent for dispersal than being large, all else being equal. More seeds in the tree's crown, positioned at more release points, should increase the probability of catching stronger than average uplifting winds needed for extended downwind dispersal (>50 m, **Table 3**), and especially turbulent wind flows needed to travel very far (LDD) [46]. This effect is further amplified by having a broader crown in LM trees. This interpretation of a synergism between a larger tree size and a larger seed crop was supported by the results of the two-dimensional model fitting of a distance pdf to the data (**Fig. 3**), which should have removed any fecundity influence on dispersal. For example, in a temperate forest, interspecific dispersal distance was positively correlated with estimates of fecundity for 14 canopy tree species [16]. An interesting, largely unexplored issue is whether ontogenetic shifts occur in allocation to reproduction in mahogany (Norghauer, *pers. observ.*) or other tree species, towards producing more smaller sized seeds on average so as to increase overall size of the fruit crop, and thereby gain a potential dispersal advantage.

### Tree height versus crown area influence on dispersal patterns

Tree crown area better explained the variation in seed dispersal than height. Tree height variation may be more important in wetter forests [57] with taller canopies than for the emergent mahogany, whose crowns are generally well exposed to winds once a reproductive diameter is reached [31] (Norghauer, *pers. observ.*). Nevertheless, tree height may matter more for explaining dispersal differences between species and life forms. For example, it was a positive factor explaining dispersal distances among nine wind-dispersed species in Panama, and all species in general [23].

Another advantage to using crown area instead of tree height is that it accounts for reductions in leaf area due to senescence or limb breakage caused by falling neighbours. Trees with a higher ratio of crown area to diameter may have more photosynthate and other resources left over after maintenance costs to invest in reproduction [34], [53]. This may partly explain the greater contribution of crown area as a strong predictor of mahogany's seed shadow extent across the 25 isolated trees.

### Modelling dispersal

Evaluating several dispersal functions was necessary because these mathematical constructions behave differently across dispersal distances. Overall the Student-*t* (2Dt) function fit best for three of the four size-fecundity groups. Compared to NE and IP, the 2Dt is more flexible at modeling copious seedfall near the parent tree and diminishing events farther downwind [20]. From a conservation perspective, whether the seed shadow tail is “thin” or “fat” has important implications for population spread and recovery in suitable habitat patches, and migration rates under climate change [6], [20]. For the wind-dispersed *Pinus halpensis*, the negative exponential (NE) consistently underestimated seed densities at <25 m distances, whereas the inverse power law did so at 25–50 m [51]. In our analysis, the inverse power (IP) behaved similarly but overestimated mahogany dispersal >50 m at trees with many fruits (LM, SM), while the negative exponential (NE) was an intermediate fit in all cases, except for LM. Elsewhere, in Mexico, the NE was deemed sufficient for describing seed dispersal downwind of small and large heavily fruiting trees, although a linear fit was better for adult trees ≥75 cm diam [38]. Elsewhere, the Student-*t* and the Gaussian generally fit best for wind-dispersed African trees species as a group [22]. This agrees with our finding for the Student-*t*. How would the 2Dt and NE fit seed density data from mahogany trees >100 cm diam? Our results suggest that either function may prove suitable for these larger-sized trees surviving only in unlogged forests. Similarly, both the inverse Gaussian (IG) and Weibull (WB) are likely suitable for modelling relative frequency of mahogany dispersal events at other sites. A future, more powerful approach will be to develop and test a mechanistic model that predicts mahogany dispersal based on seed and tree attributes and local wind characteristics (speed, direction and turbulence) [2], [14], [46].

### Mahogany juveniles, enemy escape, and LDD

Changes in seed density or abundance via dispersal play a central role in the Janzen-Connell model through their interaction with enhanced survivorship and growth rates at greater distances [10], [11], [13]. While patterns of seed density can vary within a population, as demonstrated here, a peak in the probability of adult recruitment some distance away from parent sources is nonetheless predicted (the ‘population recruitment curve’ or PRC *sensu* [10]; [58], [59]). As an example, for a widespread Mediterranean pine, the sapling-to-seed ratio peaked at 35–50 m and was lowest near source trees (0–5 m) [58]. Similarly, we found fewer mahogany juveniles near parents relative to the number of seeds landing there, suggesting these two life-stages become discordant over time [12], [58]–[60]. Indirect evidence for PRC can be seen in the discernable peak in the juvenile-to-seed ratio at 35–50 m dispersal distances (**Fig. 4**). This result was anticipated based on both observational and experimental work that showed a specialist defoliator, *Steniscadia poliophaea* (Lepidoptera: Noctuidae), targeting newly germinating seedlings near (<30 m) parent mahogany trees, and that seedling escape and survival is highest between 50–100 m from parent trees [37], [61].

The long-sought linkage between seed dispersal and plant demography remains elusive and muddled [3], [15]. But the Janzen-Connell model implicitly suggests such a linkage, because the distance at which PRC is most pronounced for a species should set a minimum intraspecific spacing for recruitment of one or possibly several adults. For this reason, LM individuals should contribute disproportionately to local population growth, as suggested by **Fig. 5**. This result reflects the reality that, in moving further downwind of parent trees, not only does the likelihood of escaping lethal attacks to new and established seedlings increase, but as the seed shadow broadens, so does the *total* area of disturbed forest in a gap-like state available for seed arrivals [12], [62].

Detecting and quantifying long-distance dispersal (LDD) is challenging because it is considered a rare event [46]. Nevertheless, it remains crucial to investigate how fecundity affects the occurrence of LDD and how, in turn, LDD may affect rates of seed survival through later stages of development [14]. The seed density vs. distance relationship for LM suggests that fecundity is vital to LDD — an interpretation strengthened by the two-dimensional results (compare LM and LF panels) — and agrees with an earlier study at Marajoara in which a few seeds dispersed at least 250 m [31]. The latter is consistent with the results from our extended sampling 100–160 m downwind, which indicate that seed dispersal by individuals of this species may form a very long dispersal tail. Longer tails and broader shadows are predicted for trees >100 cm diam, which were not available for sampling in this population. It is possible that we have underestimated dispersal events at farther distances because we did not sample a constant proportion of the annulus downwind of trees in the form of ‘wedges’. However, a trade-off was unavoidable between sampling search area and the number of replicate sources (n = 25 trees) needed to investigate population-level variation in seedfall patterns. We conclude LDD events are probably more common than is generally appreciated for mahogany, and that LDD seeds may enjoy a disproportionately high recruitment rate (see [59]). Because most remnant mahogany populations are missing their large, most fecund trees, LDD should take on greater importance in logged secondary forest than at unlogged sites. Finally, apart from ‘catching’ LDD events in real time, it is also vital to follow post-dispersal seed fates in terms of survival and mortality processes [3], [14], [15].

### Implications for conservation and management of mahogany populations

We cannot hope to effectively manage and restore economically important trees populations if we do not understand how variation in their seed dispersal arises, as well as the consequences of this variation for long-term regeneration. This issue assumes greater importance in the context of distance or density-dependent responsive natural enemies. Yet many empirical studies of dispersal use too few individuals to capture realistic variation at the population level of canopy trees. The justification often given for focussing sampling efforts on trees with large seed crops is decreased measurement error [40]. On the contrary, we argue that unbiased sampling reflecting biological realism is necessary to effectively inform policy and planning. For example, individual trees with high fruit production are the minority in logged populations of mahogany [27], [30], [31] and probably for other timber and tropical tree species in both logged and unlogged forests [34], [35], [51]–[55]. The seed shadow will determine the potential area for early regeneration, and thus spatial limits to adult recruitment. A bias towards overestimating seed shadows is risked if these are inferred from studies of large, highly fecund individuals only and then applied to the rest of the population. In the present case, this would entail applying parameter estimates from NE (or IG and WB) models for LM trees to other size-fecundity groups which form the bulk of the population in any given year and place.

In Mexico and Central America, where the specialist mahogany seedling predator *Steniscadia poliophaea* has not been reported, concentrating post-harvesting silviculture in the near (<30 m) portion of the seed shadow has been advocated because this is where progeny are most abundant [38]. This management approach cannot be advocated in South America in light of results reported here. An alternative management scheme would be to locate advance regeneration and/or seed manually at far distances downwind, where escape from *S. poliophaea* herbivory is more likely, and where the broadened seed shadow covers more gaps: both factors should contribute to increased mahogany recruitment rates in logged forests [5], [62]. Such measures take on the form of ‘strategic silviculture’, targeting the subset of the population in the best position to recruit into the canopy at minimal cost. A corollary is that large areas of forest are needed for sustained yield — but conservation benefits accrue because less land is converted to pasture or agriculture.

Sorely needed are annual observations of at least 10 yrs on *individual trees* [34], [35] to provide minimally adequate records of inter-annual variation in fruit/seed production of tropical timber species. For related Meliaceae (e.g., *Cedrela*, *Khaya*, *Entandrophragma*), counts of capsules on bare crowns with binoculars, or of pericarps on the ground, is relatively straightforward. With this information in hand, foresters could identify and retain more consistent SM, but especially LM, trees during harvests, as the latter should contribute most to recruitment. Moreover, LM trees often have hollow boles anyway and thus are wasted once felled [28]–[30].

### Conclusions

Using 25 reproductive big-leaf mahogany adults spanning the range in tree diameter and seed crops found in a selectively logged population, we examined tree-level factors shaping seed dispersal patterns. An increase in either tree size or its seed crop was sufficient to move more seeds further downwind of parents; but the seed shadow was largest and frequency of long-distance dispersal events was highest when these two traits were combined in large, high fecundity trees. Our results suggest a need for explicitly considering individual tree fecundity and both stem and crown size as possible traits influencing the shape and extent of a species' seed shadow in forests.

## Supporting Information

Supporting Information S1 A table showing the annual fruit production of each of the 25 mahogany (Swietenia macrophylla) trees used in this study in 2000–2009, with brief notes.
(PDF)
