# Supplementary material for: The Importance of Tree Size and Fecundity for Wind Dispersal of Big-Leaf Mahogany
Source: PLoS One. 2011 Mar 7;6(3):e17488. doi: 10.1371/journal.pone.0017488 (PMC3049789; doi:10.1371/journal.pone.0017488)
Supplement: Supporting Information S1 — A table showing the annual fruit production of each of the 25 mahogany (Swietenia macrophylla) trees used in this study in 2000–2009, with brief notes. (PDF) [file pone.0017488.s001.pdf]

Appendix A

Swietenia macrophylla fecundity data, expressed as number of fruit capsules per isolated tree in the study sample (n = 25) over a 10-yr period at the Marajoara forest, Pará (Brazil). Year of the present study of seed dispersal was 2005.

		Year									
**Sample**	**Tree**	**2000**	**2001**	**2002**	**2003**	**2004**	**2005**	**2006**	**2007**	**2008**	**2009**
1	30701	3	8	6	3	4	23	6	11	5	1
2	31101	124	7	80	5	2	78	18	21	0	27
3	31202	9	2	7	7	2	9	17	6	3	7
4	96	50	0	109	5	4	19	8	19	14	7
5	97	52	13	16	4	2	8	1	34	13	30
6	31302	15	0	4	4	2	9	6	8	0	1
7	31001	23	18	1	8	2	48	6	88	8	60
8	88	6	2	2	1	2	15	1	2	1	3
9	134	1	1	5	9	0	12	1	10	1	7
10	110	80	73	240	3	225	6	248	21	4	19
11	111	6	7	7	44	0	27	7	9	6	3
12	109	22	0	8	0	0	2	2	2	0	1
13	87	36	0	21	11	10	15	21	23	3	2
14	83	73	4	24	4	23	8	6	55	1	0
15	130	5	2	3	12	1	52	1	18	9	9
16	40102	2	1	3	0	0	2	8	6	10	1
17	113	1	4	3	3	0	7	5	9	6	2
18	40204	3	4	1	0	0	9	2	1	0	0
19	40503	9	2	26	18	3	34	1	24	8	15
20	40303	1	0	4	2	3	13	0	2	3	1
21	41201	3	4	5	0	4	43	2	2	6	2
22	93	5	2	2	5	5	62	10	9	2	3
23	84	28	1	16	26	11	122	3	65	22	55
24	92	28	2	10	2	0	62	6	29	1	10
25	78	53	1	142	8	5	73	13	90	14	31

<u>Summary:</u>

In a given year (2000 – 2009) 4 – 11 large trees with few fruits were observed, LF (total reproductive events = 77); and 9 – 14 small trees with few fruits, SF (total events = 109). Relatively fewer cases of high fruit production were observed in the same time 10-yr period: for SM, there were 1 – 6 such trees in a given year (total events = 25) and 1 – 9 per year of large trees with many capsules, LM (total events = 39).
